# Patient and health system costs of managing pregnancy and birth-related complications in sub-Saharan Africa: a systematic review

**DOI:** 10.1186/s13561-020-00283-y

**Published:** 2020-08-15

**Authors:** Amani Thomas Mori, Peter Binyaruka, Peter Hangoma, Bjarne Robberstad, Ingvild Sandoy

**Affiliations:** 1 Centre for International Health, University of Bergen, P.O. Box 7804, 5020 Bergen, Norway; 2 Department of Global Public Health and Primary Care, Section for Ethics and Health Economics, University of Bergen, Bergen, Norway; 3 Centre for Intervention Science in Maternal and Child Health (CISMAC), University of Bergen, Bergen, Norway; 4 Department of Health System, Impact Evaluation and Policy, Ifakara Health Institute, Dar es Salaam, Tanzania; 5 Department of Health Policy and Management, School of Public Health, University of Zambia, Lusaka, Zambia

**Keywords:** Pregnancy, Birth, Maternal complications, Cost, Catastrophic health expenditure

## Abstract

**Background:**

Morbidity and mortality due to pregnancy and childbearing are high in developing countries. This study aims to estimate patient and health system costs of managing pregnancy and birth-related complications in sub-Saharan Africa.

**Methods:**

A systematic review of the literature was conducted to identify costing studies published and unpublished, from January 2000 to May 2019. The search was done in Pubmed, EMBASE, Cinahl, and Web of Science databases and grey literature. The study was registered in PROSPERO with registration No. CRD42019119316. All costs were converted to 2018 US dollars using relevant Consumer Price Indices.

**Results:**

Out of 1652 studies identified, 48 fulfilled the inclusion criteria. The included studies were of moderate to high quality. Spontaneous vaginal delivery cost patients and health systems between USD 6–52 and USD 8–73, but cesarean section costs between USD 56–377 and USD 80–562, respectively. Patient and health system costs of abortion range between USD 11–66 and USD 40–298, while post-abortion care costs between USD 21–158 and USD 46–151, respectively. The patient and health system costs for managing a case of eclampsia range between USD 52–231 and USD 123–186, while for maternal hemorrhage they range between USD 65–196 and USD 30–127, respectively. Patient cost for caring low-birth weight babies ranges between USD 38–489 while the health system cost was estimated to be USD 514.

**Conclusion:**

This is the first systematic review to compile comprehensive up-to-date patient and health system costs of managing pregnancy and birth-related complications in sub-Saharan Africa. It indicates that these costs are relatively high in this region and that patient costs were largely catastrophic relative to a 10 % of average national per capita income.

## Introduction

An estimated 303,000 preventable deaths occurred during pregnancy and childbirth globally in 2015, mostly as a result of pregnancy and birth-related complications. Most of these maternal deaths occurred in low-income countries, particularly in sub-Saharan Africa. About three-quarters of these complications include unsafe abortions, hypertensive disorders in pregnancy i.e. pre-eclampsia and eclampsia, sepsis, severe bleeding, and complications arising at the time of delivery [1, 2]. Globally, about 17 million girls aged less than 19 years give births every year, and about 4 million undergo unsafe abortions to terminate unwanted pregnancies, and these adolescent pregnancies are associated with elevated risks of complications [1–3]. Pregnancy and childbearing complications are ranked fourth globally and second in low- and middle-income countries among the leading causes of death in adolescent girls [4].

The United Nations Development Fund reports that the prevalence of adolescent pregnancy has decreased globally, but remained relatively unchanged in sub-Saharan Africa [5]. By 2030, it is expected that the population of adolescent girls in sub-Saharan Africa will grow by 50%; hence, escalating the problem of teen pregnancy and childbearing [5]. Since adolescent pregnancy is associated with elevated risk of complications [1–3], it is likely that the total costs of treating pregnancy and childbearing complications in sub-Saharan Africa will also increase. The cost of pregnancy and birth-related complications have been synthesized and documented in systematic reviews conducted elsewhere [6–8] but not in sub-Saharan Africa despite being the region that carries the largest burden of maternal death globally [5].

This study aims to assess patient and health system costs associated with the management of pregnancy and birth-related complications in sub-Saharan Africa. The results will feed into a cost-benefit analysis study comparing two adolescent pregnancy prevention strategies in Zambia to help policymakers to choose the strategy with the greatest potential for return on investment [9]. The study findings may also be useful to researchers and policymakers elsewhere as it aims to provide cost evidence that can facilitate economic evaluation and budget impact analyses of maternal and child health interventions to demonstrate whether they represent value for money or not in addition to positive public health impact.

## Methods

We used the PRISMA checklist that is recommended for reporting a systematic review and meta-analysis of clinical trials [10], with slight modifications to suit the review of costing studies. The study protocol was registered with PROSPERO-the International Prospective Register of Systematic Reviews with registration No. CRD42019119316.

### Search strategy and inclusion criteria

The search of the literature was conducted by ATM in Pubmed, EMBASE, Cinahl, and Web of Science databases using combinations of the following search terms: cost, costs, cost of illness, economic burden, cost analysis, healthcare costs, health care costs, preterm birth, low birth weight, preeclampsia, eclampsia, abortion, post-abortion complication, cesarean section, and individual names of sub-Saharan African countries. An example of a search code used to search in Pubmed is shown in Table 1. The last search of these databases was conducted on 26th November 2018. However, we allowed Pubmed and Web of Science to send us weekly updates on the saved search terms until 13th May 2019, during which two more qualifying articles were found. Other articles were identified by scanning reference lists of review papers and relevant costing studies and searching with the Google search engine using the above-mentioned search terms. We also contacted some authors to ask for unpublished articles.

**Table 1:** Search in PubMed

No.	Search query
#1	(cost) OR “economic cost”) OR “economic analysis”) OR “economic burden”) OR “healthcare cost”) OR “cost of illness”) OR “health care cost”) OR “patient cost”))
#2	(eclampsia) OR preeclampsia) OR pre-eclampsia) OR “pre eclampsia”) OR “preterm birth”) OR “pre-term birth”) OR premature) OR “low birth weight”) OR low-birth weight) OR “lowbirth weight”) OR “small for gestational age”) OR still-birth) OR stillbirth) OR abortion) OR “post abortion complication”) OR c-section) OR “cesarean section”))
#3	(Angola) OR Benin) OR Botswana) OR Burkina Faso) OR Burundi) OR Cameroon) OR Cape Verde) OR Central African Republic) OR Chad) OR Comoros) OR Congo) OR Cote d’Ivoire) OR Djibouti) OR Equatorial Guinea) OR Eritrea) OR Gabon) OR Ethiopia) OR The Gambia) OR Ghana) OR Guinea) OR Guinea-Bissau) OR Kenya) OR Lesotho) OR Liberia) OR Malawi) OR Madagascar) OR Mali) OR Mauritania) OR Mauritius) OR Mozambique) OR Namibia) OR Niger) OR Nigeria) OR Rwanda) OR Reunion) OR (Sao Tome and Principles)) OR Senegal) OR Seychelles) OR Sudan) OR Sierra Leone) OR Somalia) OR South Africa) OR Swaziland) OR Tanzania) OR Togo) OR Uganda) OR Western Sahara) OR Zambia) OR Zimbabwe)
#4	#1 AND #2 AND #3

We included costing studies that i) were conducted in sub-Saharan Africa ii) published from January 2000 to 13th May 2019 iii) targeted normal delivery as well as pregnancy and birth-related complications including pre-eclampsia, eclampsia, pre-term birth, low birth weight babies, small for gestational age babies, unsafe abortion and post-abortion complications. The search was limited to humans and the English language. Review papers and reports were excluded because we were only interested in primary cost data, but they were instead used to identify other relevant studies. Two reviewers (ATM and PB) independently screened the titles and abstracts of all the articles to assess eligibility and the qualifying ones were subjected to further screening for eligibility by the two reviewers by reading the full text.

### Quality assessment

Quality assessment was conducted independently by ATM and PH. To the best of our knowledge, there is no quality assessment guideline for cost studies: hence, we developed an 8-item checklist from Drummond et al. [11], Liers et al. [12], and the Consolidated Health Economic Evaluation Reporting Standards (CHEERS) [13]. The 8 items were: i) description of the characteristics of the study population and the reasons why it was chosen; ii) the costing methodology used must be clearly reported, whether micro-costing or gross costing approach or a combination; iii) the sources used to collect resource utilization data should be reported clearly (e.g. clinical trials, administrative databases, clinical databases, medical records and published literature); iv) resource quantities should be reported or described independently from the costs, so that assessment of the measurement method is facilitated; v) the viewpoint/ perspective of the analysis such as the provider, patient and family or societal perspectives should be clearly described;vi) all costs should be adjusted to a specific price year so that the effects of inflation are removed from the cost estimation; vii) If the time horizon for estimating costs was longer than 1 year, discounting should have been performed to reflect time preferences viii) if prices were used instead of costs, they should reflect the true opportunity costs. Quality was assessed by scoring each of the items with a value of 1 if fully completed, 0.5 if not fully completed, 0 if not completed, and NA if not applicable. The quality scores were categorized as ‘low’ if ≤33%, ‘moderate’ if the score was between 33 and 66%, and ‘high’ if > 66%. Disagreements on eligibility or the quality assessments were resolved through consensus.

### Data analysis

We extracted information about the name of the primary author, year of publication, year in which the data was collected, study design, the country in which data was collected, costing perspective used, the currency used, cost information, disease condition, target population from which data was collected and the level of the healthcare facility.

Costs were categorized as health system costs if they were borne by the healthcare facility and patient costs if they were borne by the patient or caregiver. Health system costs could further be categorized as recurrent if spent on items that are used up in the course of the year such as salaries, supplies, and utilities or capital costs if spent on items that last more than a year such as buildings, furniture, and equipment. Patient costs included both direct costs and indirect costs. Direct costs were those paid in the process of seeking/accessing care and included out-of-pocket payment for treatment (registration, diagnosis, radiology, drugs, bed days, etc), transport to and from the healthcare facility, food, and other related expenses. Indirect costs were those that resulted from the loss of income as a result of not being able to engage in economically productive activities due to illness. When costs were reported separately for public, private, or non-governmental organizations, etc., we computed a simple average.

Base year costs in local currencies were first converted to US dollars (USD) using the existing exchange rate for the base years of the individual studies, before adjustment to 2018 USD using relevant US Consumer Price Indices (CPI) [14]. Annual Gross National Income (GNI) per capita was used as a proxy of household income and out of pocket patient payments that exceeded 10% of this income were assumed to constitute catastrophic health expenditure [15].

## Results

**Fig. 1:**
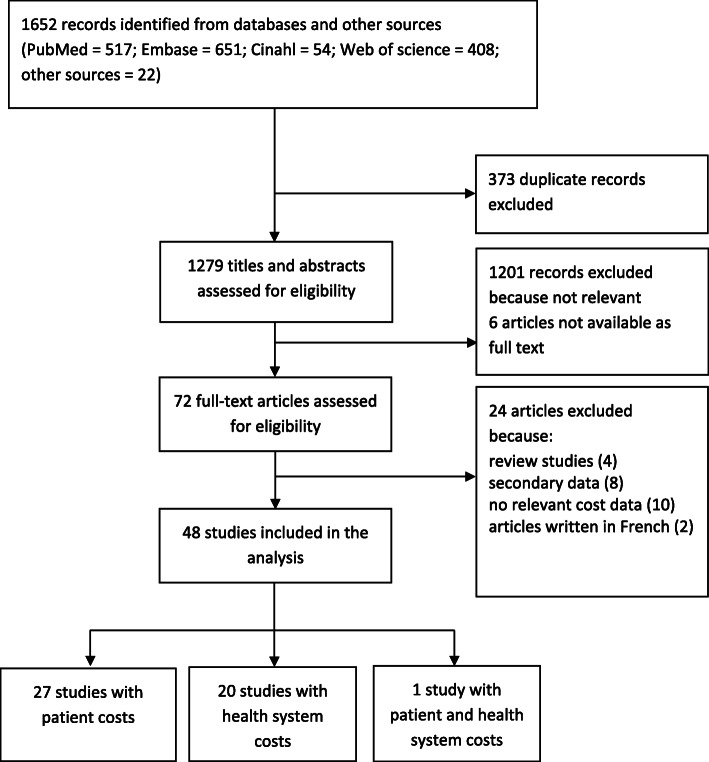
Flow diagram showing articles included and excluded in the systematic review

In total 1652 studies were identified from the systematic literature search, of which 373 studies were duplicates. The remaining 1279 unique studies were subjected to first stage screening for eligibility by reading the titles and abstracts, and as a result, 1201 studies were excluded because they were not relevant, and 6 articles were not available as full texts. The full-text screening was done for the remaining 72 articles, of which 48 were included (Fig. 1).

Table 2 shows the summary characteristics of the included studies. Most of the studies used cross-sectional design and data were collected at households and healthcare facilities depending on the chosen costing perspective. Out of the 48 studies, 36 were relatively recent and were published in the year 2010 or after. All studies were of moderate to high quality and provided a good description of the study population and the reason for its selection and the sources used to collect information about resource use. Only a few studies were explicit about the costing methodology used, but the majority provided descriptions of the perspective used.

**Table 2:** Study characteristics

Author and year	Country	Setting	Study design	Target population	Year of data collection	Quality assessment
Adamu et al. (2012) [16]	Nigeria	Urban	Cross-sectional facility based	Surviving women admitted for obstetric complications	2011	High
Akalu et al. (2012) [17]	Ethiopia	Largely rural	Cross-sectional household survey	Women (15–49 years) who have used reproductive health services in the past 12 months	2007–2008	High
Arsenault et al. (2013) [18]	Mali	Urban & Rural	Case-control and household survey	Women with obstetric emergencies	2008–2011	Moderate
Asante et al. (2007) [19]	Ghana	Unspecified	Facility survey	Women who had vaginal deliveries at health facilities, at homes and those who had C-sections	2004–2005	High
Benson et al. (2015) [20]	Malawi	Urban & Rural	Cross-sectional facility survey	Women with unsafe abortion complications	2010	High
Borghi et al. (2003) [21]	Benin	Unspecified	Cross-sectional hospital-based	Women with spontaneous vaginal delivery and near-miss obstetric complications	2000	High
	Ghana	Unspecified	Cross-sectional hospital-based	Women with spontaneous vaginal delivery and near-miss obstetric complications	1999–2000	High
Both et al. (2007) [22]	Tanzania	Urban	Cross-sectional hospital-based	Women receiving maternal healthcare services	2007	High
Carnelissen et al. (2017) [23]	Malawi	Unspecified	Cross-sectional hospital-based	Patients including women requiring a surgical procedure	2014–2015	High
Dalaba et al. (2013) [24]	Ghana	Unspecified	Cross-sectional hospital-based	Women receiving antenatal and delivery services	2010	High
Dalaba et al. (2015) [25]	Ghana	Unspecified	Cross-sectional household survey	Women with pregnancy-related complications	2014	High
Deboutte et al. (2013) [26]	DR Congo	Unspecified	Cross-sectional hospital based	Women seeking pregnancy and obstetric care	2007–2008	Moderate
Deboutte et al. (2015) [27]	DR Congo	Urban and Rural	Case-Control	Women with Caesarean section and vaginal delivery in public facilities	2007–2008	Moderate
Enweronu-Laryea et al. (2018) [28]	Ghana	Urban	Cross-sectional hospital-based	Newborns hospitalized with birth-associated brain injury and preterm/low birth weight	2016	High
Henshaw et al. (2008) [29]	Nigeria	Urban &Rural	Cross-sectional hospital-based	Women admitted to hospital for complications of induced or spontaneous abortion or to obtain an abortion	2002–2003	Moderate
Honda et al. (2011) [30]	Madagascar	Mainly urban	Cross-sectional hospital-based	Women having C-sections and children admitted for neonatal care	2007–2008	High
Ilboudo et al. (2015) [31]	Burkina Faso	Urban	Cross-sectional hospital-based	Women with induced or spontaneous abortions	2012	High
Ilboudo et al. (2016) [32]	Burkina Faso	Urban	Cross-sectional hospital-based	Women with induced abortion complications	2010	High
Johns et al. (2019) [33]	Uganda Zambia	Unspecified	Retrospective	Women attending health facilities for maternal and newborn healthcare services	2017–2018	High
Kalu-Umeh et al. (2013) [34]	Nigeria	Semi-rural	Cross-sectional community based	Women within the reproductive age group who had experienced childbirth 12 months or less before the study.	2010	Moderate
Kowalewski et al. (2002) [35]	Tanzania	Urban and Rural	Cross-sectional hospital-based	Women receiving antenatal and maternal healthcare services	1997–1998	High
Kruk et al. (2008) [36]	Tanzania	Rural	Retrospective	Women who delivered in health facilities within the previous 5 year	2007	Moderate
Le et al. (2015) [37]	South Africa	Unspecified	Cross-sectional hospital-based	Women with unintended pregnancies	2014	Moderate
Levin et al. (2000) [38]	Uganda Malawi Ghana	Unspecified	Cross-sectional hospital-based	Women presenting in healthcare facilities for maternal health services	1998	Moderate
Lince et al. (2015) [39]	South Africa	Urban	Cross-sectional hospital-based	Women accessing 2nd trimester abortion services	2010	High
Lince et al. (2018) [40]	South Africa	Urban	Cross-sectional hospital based	Women accessing 2nd trimester abortion services	2013–2014	High
Lince et al. (2017) [41]	South Africa	Urban	Cross-sectional hospital based	Women accessing 1st trimester abortion services	2009–2011	High
Lince et al. (2017) [42]	South Africa	Urban	Cross-sectional hospital-based	Women accessing 1st trimester abortion services	2011–2013	High
Lofgren et al. (2015) [43]	Uganda	Rural/Semi-urban	Prospective observational	Patients including women requiring a surgical procedure	2011	High
Meda et al. (2019) [44]	Burkina Faso	Urban and Rural	Cross-sectional hospital-based	Women who had delivered or received emergency obstetric care at public health facilities	2016	High
Ministry of Health [45]	Kenya	Urban and Rural	Cross-sectional facility survey	Women treated for unsafe abortion complications	2016	High
Moore et al. (2018) [46]	Zambia	Urban	Cross-sectional hospital-based	Women receiving safe and unsafe abortions	2014–2015	Moderate
Ntambue et al. (2018) [47]	DRC Congo	Urban	Cross-sectional hospital-based	Women receiving services in maternity wards	2014	High
Odhiambo et al. (2019) [48]	Rwanda	Rural	Retrospective	Women who delivered by emergency cesarean section	2015	High
Orach et al. (2007) [49]	Uganda	Rural	Cross-sectional hospital-based	Women receiving reproductive health services	2003	Moderate
Parmar et al. (2017) [50]	Zambia	Urban	Cross-sectional hospital-based	Women receiving safe and unsafe abortions	2013–2014	Moderate
Paul et al. (2015) [51]	Sierra Leone	Urban and Rural	Cross-sectional	Women with unsafe abortion complications	2012	Moderate
Pearson et al. (2011) [52]	Ethiopia	Urban and Rural	Cross-sectional hospital-based	Women receiving maternity services	2008–2009	Moderate
Perkins et al. (2009) [53]	Burkina Faso Kenya Tanzania	Predominantly Rural	Cross-sectional household survey	Women receiving maternity services	2006	Moderate
Ravit et al. (2015) [54]	Mali	Unspecified	Case-control	Women who underwent Caesarean section	2008–2011	Moderate
Ridde et al. (2012) [55]	Burkina Faso	Rural	Cross-sectional household survey	Women with vaginal (normal) delivery	2010	Moderate
Sambo et al. (2013) [56]	Nigeria	Rural	Cross-sectional household survey	Pregnant women and those who delivered recently (within 6 weeks postpartum)	2011	High
Sicuri et al. (2011) [57]	Mozambique	Rural	Cross-sectional hospital-based	Low birth weight babies	2007	High
Sundaram et al. (2013) [58]	Uganda	Urban and Rural	Cross-sectional household survey	Women who received post-abortion care	2011–2012	High
Tongo et al. (2009) [59]	Nigeria	Urban	Cross-sectional hospital-based	Pre term/Low birth weight neonates	2008	High
Vlassoff et al. (2012) [60]	Ethiopia	Urban and Rural	Cross-sectional hospital-based	Women who received post-abortion care	2008	High
Vlassoff et al. (2014) [61]	Uganda	Urban and Rural	Cross-sectional hospital-based	Women who received post-abortion care	2010	High
Vlassoff et al. (2015) [62]	Rwanda	Urban and Rural	Cross-sectional hospital-based	Women who received post-abortion care	2012	High
Witter et al. (2010) [63]	Senegal	Urban and Rural	Cross-sectional hospital-based	Women receiving Caesarean section and those with normal delivery	2006–2007	Moderate

**Fig. 2:**
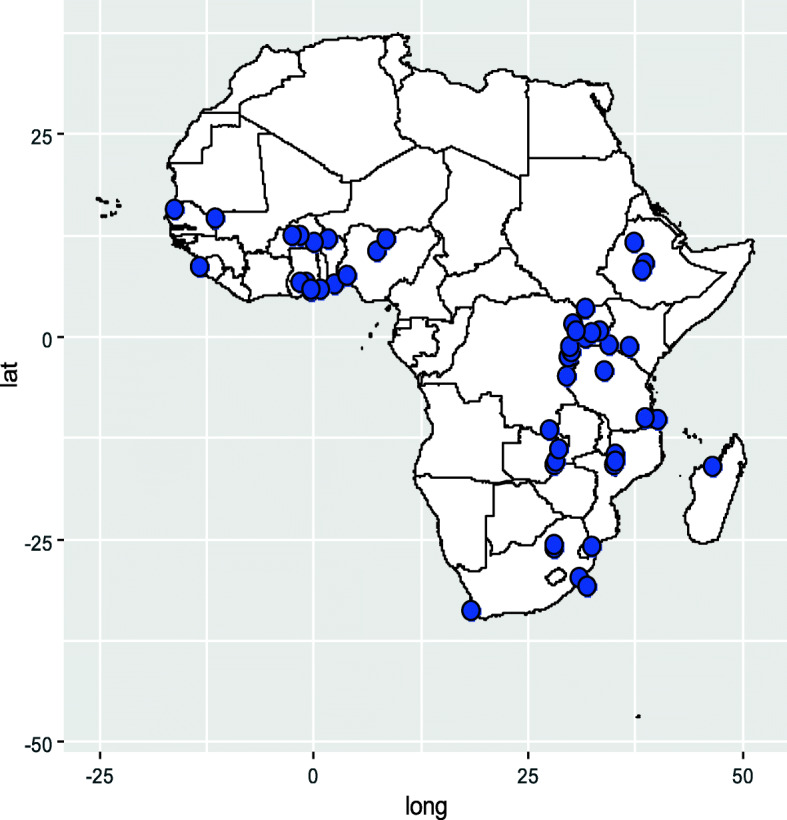
Distribution of studies in sub-Saharan Africa

Figure 2 shows the distribution of the studies in the sub-Saharan African region. The majority were from West and East Africa, while a few were from Southern Africa.

Table 3 shows the unit costs for normal delivery and C-section services. There were 19 studies from 27 countries that reported the costs of normal delivery and 20 studies from 24 countries that reported the costs of C-section. Patient cost (*n* = 13) for normal delivery range from USD 5.6–52.4 and the health system cost (*n* = 6) range from USD 8.4–72.8. However, only five of the thirteen studies reported both direct and indirect patient costs and four of the six studies reported both recurrent and capital health system costs. The patient cost (*n* = 11) for C-section ranges from USD 55.8–377.3 but only three of the eleven studies reported both direct and indirect patient costs. The health system cost (*n* = 9) for C-section ranges from USD 79.7–561.8 but only seven of the nine studies reported both recurrent and capital health system costs.

**Table 3:** Costs for normal delivery and Caesarean sections

Authors name	Country	Data collection year	Cost category	Base year cost (USD)	Cost (USD) in 2018
**Normal delivery**					
***Patient perspective***					
Asante et al. (2007) [19]	Ghana	2004	Direct	42.1	52.4
Borghi et al. (2003) [21]	Benin	2000	Direct	23.0	33.5
	Ghana	1999–2000	Direct	15.0	21.9
Deboutte et al. (2015) [27]	DR Congo	2007–2008	Direct	15.3	18.5
Kalu-Umeh et al. (2013) [34]	Nigeria	2010	Direct	9.0	10.4
Kowalewski (2002) [35]	Tanzania	1997–1998	Direct & indirect	18.5	28.5
Kruk et al. (2008) [36]	Tanzania	2007	Direct	6.9	8.6
Levin et al. (2000) [38]	Uganda	1998	Direct & indirect	17.0	26.2
	Malawi	1998	Direct & indirect	7.8	120
	Ghana	1998	Direct & indirect	16.6	25.5
Meda et al. (2019) [44]	Burkina Faso	2016	Direct	6.1	6.4
Ntambue et al. (2018) [47]	DR Congo	2014	Direct & indirect	45.0	50.2
Pearson et al. (2011) [52]	Ethiopia	2008–2009	Direct	14.4	16.8
	Tanzania	2006	Direct	4.5	5.6
	Burkina Faso	2006	Direct	6.6	8.2
Perkins et al. (2009) [53]	Kenya	2006	Direct	14.2	17.7
Ridde et al. (2012) [55]	Burkina Faso	2010	Direct	9.9	11.4
Sambo et al. (2013) [56]	Nigeria	2013	Direct	9.6	10.7
***Provider perspective***					
Both et al. (2007) [22]	Tanzania	2003	Recurrent & capital	6.3	8.6
Dalaba et al. (2013) [24]	Ghana	2010	Recurrent & capital	63.2	72.8
Johns et al. (2019) [33]	Uganda	2017–2018	Recurrent & capital	41.3	43.2
	Zambia	2017–2018	Recurrent & capital	23.0	24.1
Levin at al (2000) [38]	Uganda	1998	Recurrent	21.2	32.7
	Malawi	1998	Recurrent	14.3	22.0
	Ghana	1998	Recurrent	10.8	16.7
Orach et al. (2007) [49]	Uganda	2003	Recurrent & capital	6.1	8.4
Witter et al. (2010) [63]	Senegal	2006–2007	Recurrent	15.0	18.7
**C-section**					
***Patient perspective***					
Arsenault et al. (2013) [18]	Mali	2008–2011	Direct	107.0^*^	119.5
Asante et al. (2007) [19]	Ghana	2004	Direct	195.0	242.9
Deboutte et al. (2015) [27]	DR Congo	2007–2008	Direct	79.7	96.5
Honda et al. (2011) [30]	Madagascar	2007–2008	Direct	139.0	162.1
Kalu-Umeh et al. (2013) [34]	Nigeria	2010	Direct	99.0	114.0
Kowalewski [35]	Tanzania	1997–1998	Direct & indirect	135.0	208.0
Levin et al. (2000) [38]	Uganda	1998	Direct & indirect	36.2	55.8
	Ghana	1998	Direct & indirect	104.0	160.2
Meda et al. (2019) [44]	Burkina Faso	2016	Direct	136.4	142.7
Ntambue et al. (2018) [47]	DR Congo	2014	Direct & indirect	338.0	377.3
Pearson et al. (2011) [52]	Ethiopia	2008–2009	Direct	51.1	59.6
Ravit et al. (2015) [54]	Mali	2008–2011	Direct	163.0	182.0
***Provider perspective***					
Both et al. (2007) [22]	Tanzania	2003	Recurrent & capital	69.3	94.5
Cornelissen et al. (2017) [23]	Malawi	2014–2015	Recurrent & capital	351.0	391.8
Deboutte et al. (2013) [26]	DR Congo	2007–2008	Recurrent & capital	157.8	184.0
Johns et al. (2019) [33]	Uganda	2017–2018	Recurrent & capital	238.5	249.5
	Zambia	2017–2018	Recurrent & capital	537	561.8
Levin et al. (2000) [38]	Uganda	1998	Recurrent	79.8	122.9
	Malawi	1998	Recurrent	81.9	126.1
	Ghana	1998	Recurrent	72.2	111.2
Lofgren et al. (2015) [43]	Uganda	2011	Recurrent & capital	71.4	79.7
Odhiambo et al. (2019) [48]	Rwanda	2015	Recurrent & capital	339	359.2
Orach et al. (2007) [49]	Uganda	2003	Recurrent & capital	58.7	80.1
Witter et al. (2010) [63]	Senegal	2006–2007	Recurrent	137.0	165.9

^*^represents costs of treatment only

Table 4 shows the unit costs for abortion and post-abortion care services (PAC). There were 9 studies from 8 countries that reported the costs of abortion and 4 that reported the costs of PAC. Cost of abortion care represented mostly the medical abortion, while costs of PAC represented unsafe abortions (complete or incomplete), often performed outside the hospital setting with the woman ending up in hospital as a result of complications. Patient cost (*n* = 8) and health system costs for abortion care services (*n* = 4) range from USD 11.2–65.7 and USD 40.3–298.3, respectively. Only two of the eight studies reported direct and indirect patient costs while three of the four studies reported both recurrent and capital health system costs. For PAC services the reported patient cost (*n* = 6) ranges from USD 20.8–158.4 and all studies reported direct costs only. The health system costs for PAC (*n* = 8) were in the range between USD 46.1–151.1, and three of the eight studies reported both recurrent and capital costs.

**Table 4:** Costs for abortion and PAC

Authors name	Country	Data collection year	Cost category	Base cost (USD)	Cost (USD) in 2018
**Abortion**					
***Patient perspective***					
Akalu et al. (2012) [17]	Ethiopia	2007–2008	Direct	13.4^a^	15.7
Henshaw et al. (2008) [29]	Nigeria	2002–2003	Direct	32.2	43.9
Ilboudo et al. (2015) [31]	Burkina Faso	2012	Direct	56.0^b^	61.3
	Burkina Faso	2012	Direct	37.0^c^	40.5
Lince et al. (2015) [39]	South Africa	2010	Direct & indirect	21.2	24.5
Lince et al. (2017) [41, 42]	South Africa	2009–2011	Direct & indirect	10.0	11.2
Moore et al. (2018) [46]	Zambia	2014–2015	Direct	62.0	65.7
Pearson et al. (2011) [52]	Ethiopia	2008–2009	Direct	10.0	11.7
Sundaram et al. (2013) [58]	Uganda	2011–2012	Direct	23.0	25.2
***Provider perspective***					
Le et al. (2015) [37]	South Africa	2014	Recurrent & capital	281.2	298.3
Lince et al. (2017) [41, 42]	South Africa	2011–2013	Recurrent & capital	65.4	70.5
Lince et al. (2018) [40]	South Africa	2013–2014	Recurrent & capital	250.3^d^	265.5
Parmar D [50]	Zambia	2013–2014	Recurrent	38.0^e^	40.3
**Post-abortion care**					
***Patient perspective***					
Henshaw et al. (2008) [29]	Nigeria	2002–2003	Direct	116.0	158.4
Ilboudo et al. (2015) [31]	Burkina Faso	2012	Direct	33.0^b^	36.1
	Burkina Faso	2012	Direct	19.0^c^	20.8
Meda et al. (2019) [44]	Burkina Faso	2016	Direct	32.1	33.6
Moore et al. (2018) [46]	Zambia	2014–2015	Direct	81.0	85.8
Sundaram et al. (2013) [58]	Uganda	2011–2012	Direct	26.0	28.4
***Provider perspective***					
Benson et al. (2015) [20]	Malawi	2010	Recurrent	40.0	46.1
Levin et al. (2000) [38]	Uganda	1998	Recurrent	46.5	71.7
	Malawi	1998	Recurrent	35.9	55.2
	Ghana	1998	Recurrent	65.2	100.4
MoH –Kenya (2018) [45]	Kenya	2016	Recurrent	58.0	60.7
Parmar et al. (2017) [50]	Zambia	2013–2014	Recurrent	52.0	55.2
Paul et al. (2015) [51]	Sierra Leone	2012	Recurrent	68.0	74.4
Vlassoff et al. (2014) [61]	Rwanda	2012	Recurrent & capital	93.0	101.7
Vlassoff et al. (2012) [60]	Uganda	2010	Recurrent & capital	131.2	151.1
Vlassoff et al. (2009) [64]	Ethiopia	2008	Recurrent & capital	100.0	116.6

^a^average of costs in public facilities (USD 16.12) and private USD 10.73

^b^procedure for induced abortion

^c^cost of care for spontaneous abortion

^d^average for dilatation & evacuation with misoprostol (88.90 USD), medical induction with mifepristone+misoprostol (298.03 USD) and medical induction with misoprostol only (364.08 USD)

^e^ Costs of unsafe abortion

Table 5 shows the unit costs for the management of eclampsia, low birth weight, and hemorrhage. There were 17 unique studies conducted in 22 countries. For the management of eclampsia, patient costs (*n* = 5) range between USD 51.6–230.5, and two of the five studies reported both direct and indirect costs. The health system costs for eclampsia (*n* = 2) range from USD 122.7–186.4 and no capital cost was measured. For care of low-birth weight babies, the patient cost (*n* = 3) ranged between USD 38.2–486.7, and two studies contained both direct and indirect costs, while only one study by Sicuri et al. (2011) from Mozambique reported an average health system cost of about USD 514 for caring such babies. For the management of maternal hemorrhage, patient cost (*n* = 4) ranges between USD 65.1–196.2, and half of the studies reported both direct and indirect costs. The health system cost for maternal hemorrhage range between USD 30.3–127.4 and all the studies reported recurrent health system cost only.

**Table 5:** Costs of other complications

Authors name	Country	Data collection year	Cost category	Base cost (USD)	Cost (USD) in 2018
**Eclampsia**					
***Patient perspective***					
Borghi et al. (2003) [21]	Benin	2000	Direct	119.0	173.5
	Ghana	1999–2000	Direct	69.0	100.6
Dalaba et al. (2015) [25]	Ghana	2014	Direct & indirect	58.3	61.9
Meda et al. (2019) [44]	Burkina Faso	2016	Direct	49.3	51.6
Ntambue et al. (2018) [47]	DR Congo	2014	Direct & indirect	206.5^b^	230.5
Ravit et al. (2015) [54]	Mali	2008–2011	Direct	179.8	200.7
***Provider perspective***					
Levin et al. (2000) [38]	Uganda	1998	Recurrent	121.015	186.4
	Malawi	1998	Recurrent	79.62	122.7
**Low birth weight babies**^a^					
***Patient perspective***					
Enweronu et al. (2018) [28]	Ghana	2016	Direct & indirect	147.6	154.4
Sicuri et al. (2011) [57]	Mozambique	2007	Direct & indirect	31.5	38.2
Tongo et al. (2008) [59]	Nigeria	2008	Direct	417.3	486.7
***Provider perspective***					
Sicuri et al. (2011) [57]	Mozambique	2007	Recurrent & capital	424.6	514.2
**Hemorrhage**					
***Patient perspective***					
Borghi et al. (2003) [21]	Benin	2000	Direct	104.0	151.7
	Ghana	1999–2000	Direct	79.0	115.2
Dalaba et al. (2015) [25]	Ghana	2014	Direct & indirect	6.84	7.3
Meda et al. (2019) [44]	Burkina Faso	2016	Direct	58.35	65.2
Ntambue et al. (2018) [47]	DR Congo	2014	Direct & indirect	187.5^b^	196.17
Ravit et al. (2015) [54]	Mali	2008–2011	Direct	140.34	156.67
***Provider perspective***					
Ilboudo et al. (2016) [32]	Burkina Faso	2010	Recurrent	26.3	30.3
Levin et al. (2000) [38]	Uganda	1998	Recurrent	82.7	127.4
	Malawi	1998	Recurrent	74.3	114.5
	Ghana	1998	Recurrent	65.3	100.5

^a^Costs from delivery to discharge from hospital

^b^Average cost for vaginal and c-section

**Fig. 3:**
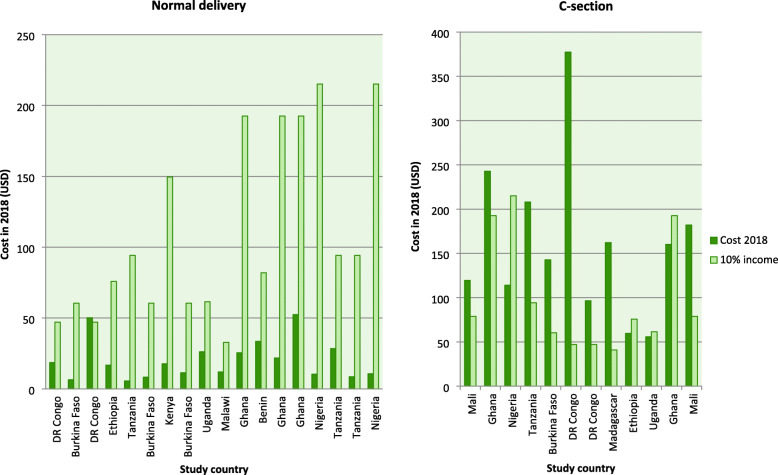
Out of pocket cost versus 10% of per capita income for normal vaginal delivery and C-section

**Fig. 4:**
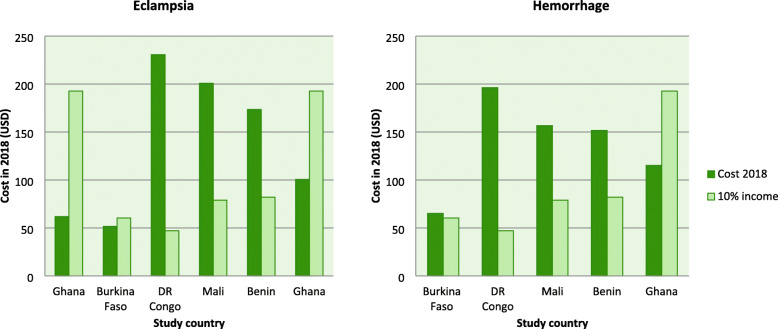
Out of pocket cost versus 10% of per capita income for specific birth complications

Figures 3 and 4 compare whether out of pocket health expenditures for normal delivery, C-section, eclampsia, and maternal hemorrhage was higher than 10% of the average gross national income per capita for different countries in sub-Saharan Africa. Out of pocket cost for normal delivery services was catastrophic for only one study from DR Congo [47], however, costs were catastrophic in eight studies out of the twelve [18, 19, 27, 30, 35, 44, 47, 54] that reported delivery by the C-section. Out of pocket payments were also catastrophic in three out of six studies on the management of eclampsia [21, 47, 54], one out of four studies about abortion services [31], one out of three studies on the management of low birth weight babies [59] and four out of five studies on the management of hemorrhage [21, 44, 47, 54]. None of the studies on PAC costs indicated that catastrophic health expenditures were incurred.

## Discussion

This review shows that pregnancy and childbearing expose women and their families to a lot of out-of-pocket (OOP) payments, particularly in the presence of complications. For normal spontaneous vaginal deliveries, women pay between USD 5.6–52.4 and for C-section they pay between USD 55.8–377.3, meaning on average it costs seven times more to deliver by C-section. The OOP payments usually constitute costs of drugs and medical supplies like cotton wools, syringes, transportation to and from the health facilities, food, drinks and unofficial payments to health workers. Mean OOP payments were either very close to or exceeded 10 % of an average national per capita income for some countries, thus most likely exposing patients and their families to substantial financial burden.

Results from the included studies show that catastrophic health expenditures were common among the study participants [16–18, 27, 30, 31, 59]. In Bunia DR Congo, the user cost of C-section was estimated at 79.7 USD, which was slightly above the monthly family income of 75.5 USD [27]. In Birnin-Kebbi Nigeria, average monthly family income was 18.8 USD compared to the average cost of care for emergency obstetric care (EmOC) of about 246 USD [16]. In rural Ethiopia, more than two-thirds of the studied families experienced catastrophic health expenditure for maternal healthcare [17]. In Mali, between 20 and 54% of the studies households incurred catastrophic health expenditure on EmOC [18]. In Burkina Faso, 12% of women with abortion experienced catastrophic health expenditure [31]. In Madagascar, the proportion of OOP for C-section among the richest and the poor was 33% and 109%, respectively [30].

Our study indicates that pregnancy and childbearing complications are also relatively expensive to the healthcare systems in sub-Saharan Africa. Health systems use between USD 8–73 per patient for normal deliveries, but a staggering USD 80–562 for C-section, USD 40–300 for medical abortion, USD 40–150 for post-abortion care, USD 120–190 to care for eclampsia, USD 30–130 to treat hemorrhage and about USD 500 to care for low-birth weight babies. In 2009 it was estimated that the annual cost to treat unsafe abortion complications in sub-Saharan Africa ranged from USD 68–76 million [64] and in 2014, it was estimated that the cost required to provide post-abortion care in developing countries was USD 232 million [65]. A large chunk of these costs could be prevented by investing in modern contraceptive use to prevent unwanted pregnancies, legalizing abortion where it is illegal and implementing policies with the potential to reduce adolescent pregnancies. High costs that are associated with access to healthcare hinder the utilization of maternal health services in resource-poor settings [66, 67].

This study has several limitations, which requires care in its interpretation. Firstly, the included studies were methodologically very heterogeneous in terms of range patient and health system costs included making it hard to fully disaggregate the costs. Secondly, costs are very context-specific especially for non-traded goods and services such as wages and salaries, which are usually one of the main cost drivers. Thus, in countries where salaries and prices of commodities are high always tend to skew the average costs. Also, there could be a lot of variations in the structure and complexity of the healthcare system and services available for managing pregnancy and birth-related complications between countries, hence resource requirements and costs could infinitely vary from one place to another. For this reason, we could not aggregate the costs into meaningful means or medians.

Our findings regarding the costs of maternal health care have several policy implications despite the limitations. First, it is well documented that adolescent pregnancy and childbearing are associated with elevated risks of complications [68, 69], which are mainly concentrated in sub-Saharan Africa [70]. Our study enhances the understanding of the financial implications of these complications both for patients, families, and health systems. Policies that can delay teen pregnancies, therefore, have the potential not only to reduce maternal morbidity and mortality but also to save patients and health systems a significant amount of healthcare resources. Second, this review shows that maternal complications may result in OOP expenditures that are largely catastrophic especially among the poorest households. New innovative strategies are urgently needed to protect women and their families from impoverishing OOP, otherwise, the real impact of abolishing user fees for maternal services will be hard to be realized.

## Conclusion

This is the first systematic literature review to compile comprehensive up-to-date patient and health system costs of managing pregnancy and birth-related complications in sub-Saharan Africa. It indicates that these costs are relatively high. It further shows that patient costs were largely catastrophic relative to a 10 % of average national per capita income, thus exposing families to immense financial burden and impoverishment, in particularly poor families that live under one USD per day. Hence health policies that advocate for free maternal health services and universal health coverage on maternal and newborn care should be encouraged and prioritized on both national, regional, and international agenda. Otherwise, the high costs will continue to hinder access to maternal health services in sub-Saharan Africa, thus negating the efforts to reduce infant and maternal mortality rates which are relatively high in this region. Although the study found a relatively large number of studies, the evidence base on the costs of maternal care is nevertheless still scarce; hence, more studies are needed to fill the gaps.

## Data Availability

Not applicable.

## Abbreviations

C-section: Caesarean Section
EmOC: Emergency obstetric care
GNI: Gross National Income
OOP: Out of pocket
PAC: Post abortion care
USD: United States Dollar
